# Osseous Union of Teeth

**Published:** 1857-10

**Authors:** 


*Osseous Union of Teeth.*—The senior editor has frequently had occasion to notice examples of Osseous Union of Teeth, but the most remarkable specimen which he has ever seen, was presented to him at the late Convention in Boston, by Dr. H. A. Emery. It is a dens sapientiæ and a small supernumerary tooth. The side of the crown of the latter is united to the grinding surface of the crown of the former—the root of the supernumerary pointing upwards and backwards. In this most curious example of osseous union of teeth, the last mentioned tooth must have been developed from a sack given off from the coronal portion of the sack of the wisdom tooth, and a union of the enamel membrane of the two teeth must have taken place previous to the deposition of earthy salts in the cells of the enamel fibres. This, we believe, is the only way in which such an occurrence could possibly have taken place. We shall place the specimen in the Museum of the Baltimore College of Dental Surgery.

